# Synergistic *vs*. complementary synbiotics: the complexity of discriminating synbiotic concepts using a *Lactiplantibacillus plantarum* exemplary study

**DOI:** 10.20517/mrr.2024.48

**Published:** 2024-09-04

**Authors:** Michiel Kleerebezem, Jori Führen

**Affiliations:** ^1^Department of Animal Sciences, Host Microbe Interactomics Group, Wageningen university and Research, Wageningen 6708 WD, the Netherlands.; ^2^Laboratory of Food Microbiology, Wageningen university and Research, Wageningen 6708 WG, the Netherlands.

**Keywords:** Synbiotics, *Lactiplantibacillus plantarum*, prebiotics, ecosystem interaction, nutrient competition

## Abstract

Synbiotics are defined as “a mixture comprising live microorganisms and substrate(s) selectively utilized by host microorganisms that confers a health benefit on the host”. The definition discriminates between synergistic and complementary synbiotics. Synergistic synbiotics involve a direct interaction between the substrate and co-administered microbe(s), while complementary synbiotics act through independent mechanisms. Here, we evaluate the complexity of discrimination between these two synbiotic concepts using an exemplary study performed with a panel of *Lactiplantibacillus plantarum* (*L. plantarum*) strains to identify strain-specific synergistic synbiotics that eventually turned out to work via a complementary synbiotic mechanism. This study highlights that assessing the *in situ* selectivity of synergistic synbiotics in the intestinal tract is challenging due to the confounding effects of the substrate ingredient on the endogenous microbiome, thereby raising doubts about the added value of distinguishing between synergistic and complementary concepts in synbiotics.

## SYNBIOTICS

The term “synbiotic” refers to “synergy” and the Greek word for life “bios”, effectively meaning “synergistic life” and is an obvious nod to the terms prebiotic and probiotic and their potential synergy in the host’s digestive tract. Accordingly, the initial definition of synbiotics was “probiotics and prebiotics that beneficially affect the host”^[1]^. In 2020, an expert panel assembled by the International Scientific Association for Probiotics and Prebiotics (ISAPP) refined the synbiotics definition as “a mixture comprising live microorganisms and substrate(s) selectively utilized by host microorganisms that confers a health benefit on the host”. The panel discriminated between complementary and synergistic synbiotics. In complementary synbiotics, the components non-cooperatively exert their synergistic health-promoting effects in the host, whereas in synergistic synbiotics, the substrate is designed to be selectively utilized by the co-administered microorganisms to enhance their health-promoting effects^[2]^. Notably, the latter concept does not exclude enrichment of other beneficial members of the endogenous microbiota, but identifies the co-administered microbe as the main target of the prebiotic substrate. Additionally, the synergy underlying the synbiotic concept intrinsically implies that these functional foods elicit a superior effect compared to those elicited by the separate administration of their constituents. However, due to the complications in scientifically establishing a synergistic effect on health, the consensus described by the expert panel^[2]^ specifies that for synergistic synbiotics, it would be sufficient to demonstrate selective use of the prebiotic substrate by the co-administered microorganism.

Health effects associated with the consumption of synbiotics include the general improvement of anthropometric, cardiometabolic, and inflammatory markers^[3]^, as well as the eradication of *Helicobacter pylori*^[4]^ and symptom improvement in various patient groups, including those suffering from atopic dermatitis^[5]^, nonalcoholic fatty liver disease^[6]^, and colorectal cancer^[7]^. Notably, in most of these studies, it remains unclear whether consumption of the synbiotic product has an added value over the consumption of its individual ingredients [i.e., the probiotic(s) or prebiotic(s)], and it is often not specified whether the synbiotic would classify as a complementary or synergistic synbiotic. Therefore, despite the refined definition of synbiotics, most synbiotic designs (i.e., the choice of pre- and probiotics that are combined) remain poorly rationalized and their health-promoting efficacy relative to their individual constituents often remains unclear.

### Synbiotics can improve general health in newborns at risk

The most striking health impact associated with the administration of a synbiotic product has undoubtedly been described by Panigrahi *et al.*, reporting on a large-scale, double-blind, placebo-controlled randomized synbiotic administration trial (*N* > 4,500) in infants recruited in Odisha, India, achieving significantly decreased neonatal sepsis and mortality in the synbiotic group compared to the placebo group^[8]^. Additionally, the synbiotic-treated group also displayed a significantly reduced incidence of lower respiratory tract infections and diarrhea, indicating that the overall health status of the synbiotic-treated children was improved compared to the placebo group^[8]^. The synbiotic product used in this landmark study was composed of *Lactiplantibacillus plantarum* (*L. plantarum*) ATCC202195 in combination with fructo-oligosaccharides (FOS), which was administered to newborns for 7 consecutive days starting 2-4 days after birth. Both this study^[8]^ and earlier work^[9]^ by the same group established that this synbiotic administration regimen succeeded in establishing long-term colonization (several months) of the *L. plantarum* strain in the intestines of these children. However, the synergistic interaction of the individual pre- and probiotic constituents in this study remains undetermined, and it was not reported whether the ATCC202195 strain could effectively utilize FOS as a substrate for growth. Analysis of the available genome sequence of this strain (NCBI: genome/GCF_004354995.1) indicates that it lacks a gene encoding an extracellular β-fructosidase (FosE), which has been shown to be required for the utilization of longer chain fructo-oligo- and polysaccharides^[10-12]^. This finding suggests that *L. plantarum* ATCC202195 would only utilize the short-chain fraction of the FOS product used (mono-, di- and tri-saccharides; fructose, sucrose, and 1-kestose, respectively), analogous to what has been observed for other FosE-lacking strains of this species^[12,13]^. These considerations leave it undecided whether the synbiotic used in this study should be regarded as a complementary or synergistic synbiotic mixture. Moreover, due to the lack of single-constituent control interventions, it remains to be established whether the administration of only *L. plantarum* ATCC202195 (i.e., without FOS co-administration) could achieve the same health impacts.

In the section below, we will review some of the work performed with *L. plantarum* as an exemplary case for the design of strain-specific synergistic synbiotics and the exploration of their capacity to (selectively) enhance the intestinal fitness of *L. plantarum*.

## DESIGNING SYNERGISTIC SYNBIOTICS

### Strain-specific synergistic synbiotics for *L. plantarum*

Strains of the species *L. plantarum* have long been recognized as excellent probiotic candidates^[14-17]^. The species has a very wide ecological distribution ranging from the GI tract of humans and animals to decaying plant materials and fermented and non-fermented food products^[18]^. *L. plantarum* WCFS1, a single isolate of strain NCIMB8826, is among the most documented and extensively studied *L. plantarum* strains and has been instrumental in our understanding of the lifestyle of this species. The *L. plantarum* WCFS1 genome was the first genome of the lactobacilli to be published^[19]^ and has since then been extensively analyzed, including a comprehensive overview of its predicted secretome^[20,21]^, a genome-scale metabolic model^[22]^, and a reconstruction of its regulatory network^[23]^. The availability of these tools rendered the WCFS1 strain a useful model for in-depth investigation of the molecular mechanisms that underlie probiotic function in lactobacilli^[24]^. More recently, comparative genomics of 54 *L. plantarum* strains^[25]^ (later expanded to 611 genomes^[26]^) revealed a large pan-genome (> 7,000 orthologous groups), which did not approach saturation (i.e., also not with 611 genomes^[26]^), indicating that the genomic diversity of the species was still substantially larger than the 7,000 orthologous groups found in the 54 strains^[25]^, which was expanded to more than 20,000 genes in 611 strains^[26]^. These findings agreed with the ecological flexibility and nomadic lifestyle of *L. plantarum*^[25,27]^.

The genotypic and phenotypic diversity of *L. plantarum* strains is strongly reflected by the highly strain-specific carbohydrate utilization gene-repertoires, which is reflected by an array of genomic “lifestyle” islands that contain genes annotated as carbohydrate utilization cassettes, which appear to be clustered close to the origin of replication. Interestingly, no correlation between the diversity in these cassettes and the niche of isolation of these strains could be detected^[18,25,28]^. These strain-specific carbohydrate utilization gene repertoires offer an attractive starting point for the development of strain-specific synbiotics. Therefore, we developed a synbiotic matchmaking approach in our lab to identify prebiotic substrates that could differentially be utilized for growth by a panel of 77 *L. plantarum* strains^[29]^. Substantial variations in prebiotic utilization capacity were detected among the 77 strains for galacto-oligosaccharides (GOS) and isomalto-oligosaccharides (IMOS), whereas only a single strain (*L. plantarum* Lp900) isolated from ogi, a fermented sorghum pudding from Nigeria, was able to effectively utilize short- and long-chain fructo-oligosaccharides (FOS and inulin, respectively)^[29]^. Notably, it is well-established that the substrates that supported variable growth of the *L. plantarum* strains (i.e., GOS and IMOS) encompass a variety of distinct saccharide constituents that vary in degree of polymerization (DP) and glycosidic linkages^[30,31]^. Refinement of the matchmaking approach by determination of the strain-specific utilization capacity of individual GOS and IMOS constituents was able to explain the observed variations in the overall utilization (growth) of these prebiotics, which through gene-trait matching could be linked to specific *L. plantarum* genes that are involved in the metabolization of these IMOS and GOS constituents^[29,32]^. Similarly, the single *L. plantarum* strain in the panel (Lp900) that could effectively grow on FOS or inulin was shown to contain a plasmid encoding a cell-wall anchored extracellular β-fructosidase (FosE) as well as a fructose import system, facilitating effective degradation and growth on FOS and inulin^[11,12]^. Besides the prebiotic substrates mentioned above, the matchmaking study detected only marginal utilization of other candidate prebiotics like arabinoxylan oligosaccharides (AXOS) and fucoidan^[29]^. The latter finding does not exclude the possibility that expansion of the *L. plantarum* strain panel could enable the identification of strains that are able to utilize these substrates, particularly when *L. plantarum* isolates obtained from niches containing these substrates would be included.

The finding that specific IMOS and GOS constituents could only be utilized by some strains offers opportunities for further refinement of the synbiotic combinations that would more selectively stimulate specific strains. For example, IMOS preparations commonly contain a substantial amount of isomaltose (α-1,6-linked glucose-glucose disaccharide), which could be utilized by some but not all *L. plantarum* strains. This capacity to utilize isomaltose perfectly correlated with the presence of a gene cluster that was proposed to encode an (iso-)maltose phosphotransferase system (PTS), an (iso-)maltose-6’-phosphate glucosidase, a β-phosphoglucomutase, and a transcriptional regulator. Accordingly, follow-up experiments that used isomaltose as a sole carbon source for growth exclusively stimulated the growth of the subset of strains that encoded these functions^[29]^. Similarly, a subset of the strains was able to utilize the higher DP isomaltose constituents of IMOS (estimated DP > 3), which could also be associated with a gene cluster encoding several ABC-import systems annotated to import multiple sugars and several α-mannosidases, which appears in agreement with the observed higher-DP IMOS utilization phenotype, and suggests that these oligosaccharides are not degraded extracellularly but are imported and intracellularly hydrolyzed and used to support growth^[29]^. Further analysis of the GOS constituent utilization per strain revealed that the strains could predominantly be divided into two utilization groups. The minority of the strains could utilize several glucose-galactose disaccharides, including lactose (β-1,4-linked) and the β-1,2- and β-1,3- disaccharides, but could not utilize the β-1,2 and β-1,3 galactose-galactose disaccharides. The majority of the strains could utilize the latter galactose-galactose disaccharides, as well as some higher-DP oligosaccharide constituents of GOS (estimated DP > 3), to varying extents^[32]^. The strains with the extended GOS-constituent utilization capacity encoded a *lacAS* operon with a divergently oriented transcription regulator encoding gene (*lacR*) that was absent in the other group of strains. The *lacAS* operon was annotated to encode a β-galactosidase (LacA) and a GPH-family permease (LacS) that is annotated to be involved in the import of lactose and galactose. However, the introduction of a *lacS* mutant did not only result in reduced growth on lactose, but also a complete loss of the higher DP-fraction constituent utilization, confirming the role of the *lac* operon in the observed variability of the GOS-utilization phenotype^[32]^. Similar to what was found for IMOS, the results imply that the higher-DP constituents of these prebiotics are first imported into the cell to be subsequently hydrolyzed and catabolized. Moreover, for both IMOS and GOS prebiotics, further fractionation of these complex saccharide mixtures would enable the isolation of specific constituents that would more exclusively stimulate the growth of a few *L. plantarum* strains, which opens the door for precision-prebiotic substrates for highly selective synbiotic combinations that would stimulate the growth of only few specific strains.

### Establishing increased intestinal survival and persistence by prebiotic supplements

Following the identification of prebiotic substrates that could selectively stimulate the growth of specific *L. plantarum* strains, subsequent experiments aimed to verify that the prebiotic substrate inulin was able to stimulate the intestinal survival and persistence of *L. plantarum* Lp900 *in situ* in the intestine using a preclinical rat model. As mentioned above, strain Lp900 contains a plasmid encoding a cell-wall anchored extracellular β-fructosidase (FosE), as well as a fructose import system, facilitating effective degradation and growth on inulin. The results obtained clearly established that inulin supplementation of the rats’ diet significantly enhanced the intestinal delivery of *L. plantarum* Lp900 compared to rats that were fed a non-supplemented diet^[12]^. This result supports that the identified candidate synergistic synbiotics are able to stimulate the *in situ* delivery of their matched *L. plantarum* strain. In the same study, it was recognized that the stimulatory effect of inulin was much more pronounced when inulin was added to diets that had a high calcium phosphate level compared to those with a low level, indicating that the background diet, particularly its micronutrient levels, has a marked impact on the stimulatory efficacy of inulin supplementation. These results aligned with previous studies showing that high dietary calcium phosphate intake (primarily from dairy products) was associated with higher levels of endogenous lactobacilli in the intestines compared to individuals with lower calcium phosphate intake^[33]^. The mechanisms by which dietary calcium (calcium phosphate) modulates the intestinal microbiota are not fully understood, but it has been proposed to depend on the increased buffering capacity of the intestinal lumen and the ensuing precipitation of cytotoxic surfactants like secondary bile acids^[34,35]^. The latter compounds are established antimicrobials with particular effective inhibitory capacities against endogenous Gram-positive bacteria like lactobacilli^[36]^. At first sight (see also below), these results supported the synergistic mechanism of action of the synbiotic combinations identified through the *in vitro* matchmaking and gene-trait matching approach described above.

### Competition *in vitro* or *in situ* in the gut: nutrient competition or environmental selection?

The final stage of this line of research on strain-specific *L. plantarum* synbiotic combinations intended to investigate the term “selectively” in the synbiotic definition, because the inulin-mediated enhancement of the intestinal fitness of *L. plantarum* Lp900 in rats fed an inulin-containing diet did not directly assess this aspect.

**Figure 1:**
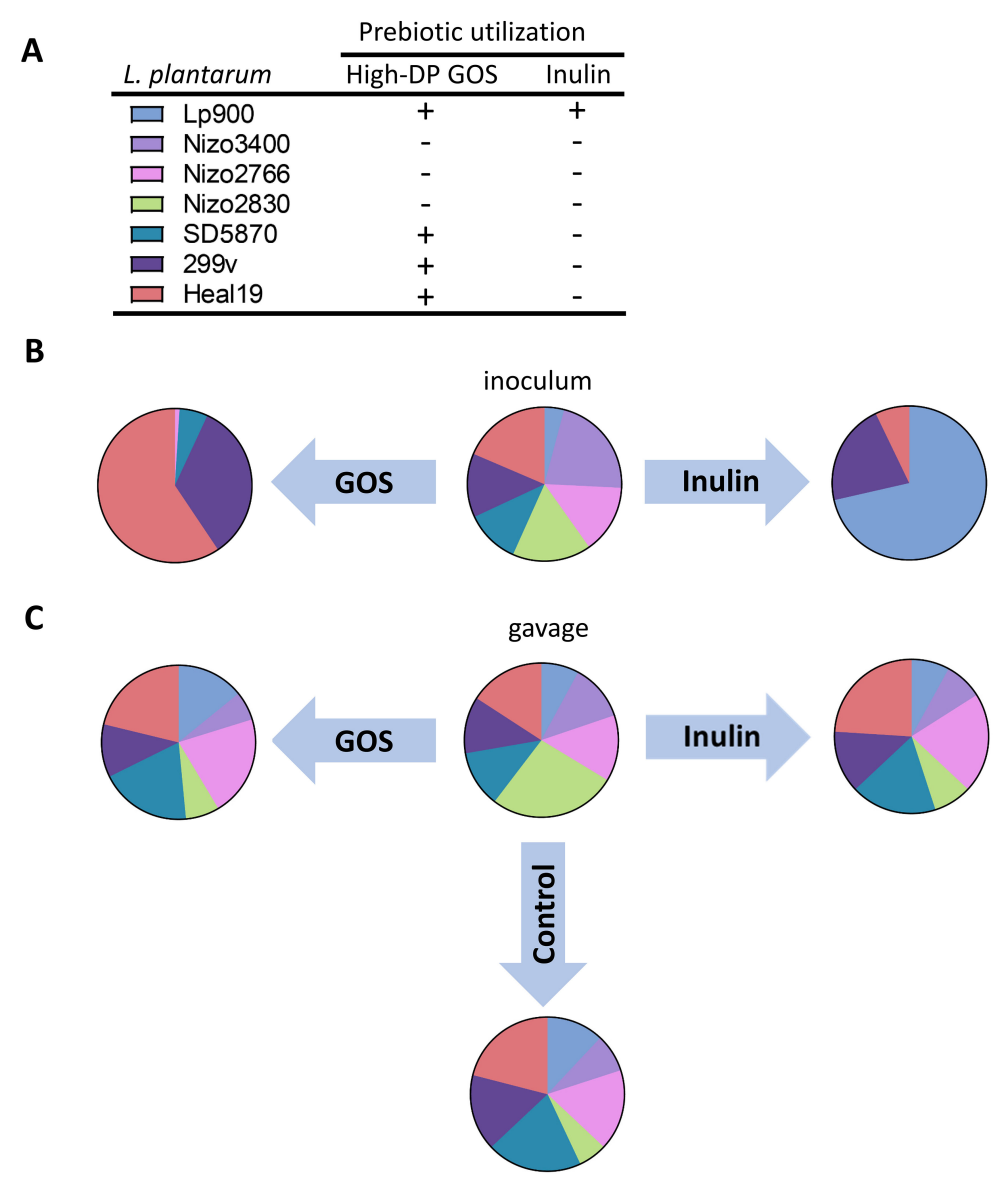
*In vitro* and *in situ* competitive fitness assessment of 7 *L. plantarum* strains. (A) The 7 *L. plantarum* strains used in this study. The strains were selected based on their discriminating prebiotic utilization phenotypes for GOS and inulin, combined with the possibility for high-throughput population dynamics based on strain-specific intergenic alleles that can be assessed using next-generation amplicon sequencing (for details, see ref^[41,42]^); (B) The strain-specific *L. plantarum* population shifts observed relative to the 7-strain inoculum mixture after 72 generations (generations are here defined as divisions of the overall seven strain population, and thus does not equal the number of generations of an individual strain in the seven strain mixture) of growth in a laboratory medium that contained GOS and inulin as a sole carbon source for growth, revealing considerable enrichment of at least some of the prebiotic utilizing *L. plantarum* strains by outcompeting non-utilizing strains; (C) The *L. plantarum* strain-specific population compositions observed in fecal samples obtained 7 days post-gavage, in comparison to the population composition of the 7-strain gavaged mixture, in rats that were fed a high-calcium control diet or the same diet supplemented with the prebiotic GOS or inulin. No significant population composition changes occurred during these 7 days in the intestinal tract, irrespective of the diet fed to the rats. *L. plantarum*: *Lactiplantibacillus plantarum*; GOS: galacto-oligosaccharides.

In the context of substrate-mediated selective fitness stimulation, it is important to realize that this is strongly influenced by the mechanism by which microbes utilize the substrate. The extracellular degradation of inulin by *L. plantarum* Lp900 is a “cooperative” trait that liberates the fructose building blocks of inulin as “public goods” in the environment of the cell. Consequently, the selectivity of inulin as a substrate for the growth of *L. plantarum* Lp900 in a microbial ecosystem may suffer from so-called “cheaters” that do not contribute to the cooperative trait of degrading the polymeric substrate, but capitalize on the availability of the public goods. In contrast, the *L. plantarum* strains that can utilize the high-DP constituents of IMOS and GOS by internalizing these substrates followed by intracellular degradation and metabolization monopolize these substrates (i.e., “privatized goods”) and circumvent cheater-risks^[37-40]^. These considerations should be taken into account when assessing substrate-induced competitive fitness advantage, as the selectivity of such advantages may depend on the substrate and the mechanism of its utilization. In view of these considerations, the relative selectivity of the fitness benefits associated with specific prebiotic utilization capacities of individual *L. plantarum* strains was evaluated using a panel of seven genetically distinguishable strains^[41,42]^, which differ in their inulin and GOS utilization capacities. Initial experiments evaluated the population dynamics of the seven *L. plantarum* strains during approximately 72 generations of *in vitro* growth in media containing inulin or GOS as a sole carbon source (Figure 1, adapted from ref^[41]^). These simple *in vitro* competition experiments revealed that the growth of the mixture of strains on both GOS and inulin as a substrate led to a significant enrichment of the strains that are able to utilize these substrates^[41]^. Notably, strain *L. plantarum* Lp900 (the inulin-degrading strain) clearly accumulated in the population after 72 generations of growth on inulin, but this coincided with the enrichment of two potential cheater strains (299v and Heal19). Additionally, after 72 generations of growth on GOS, two of the strains - 299v and Heal19 - that could import and utilize High-DP GOS constituents showed significant enrichment. In contrast, the remaining two strains displayed modest (SD5870) or no (Lp900) enrichment under those conditions. These observations support that the prebiotic-matchmaking results are able to at least partially predict the selective fitness advantage of these *L. plantarum* strains during *in vitro* growth in a simple microbial consortium of strains of the same species. Notably, *L. plantarum* 299v and Heal19 were consistently among the most robust growing strains on various substrates, which may explain their success as cheaters in the inulin cultures and their apparent competitive advantage over SD5870 and Lp900 in High-DP GOS nutrient competition [Figure 1].

Selective fitness advantages are further complicated when assessed in a complex microbial ecosystem such as the gut microbiome, where substantial redundancy for prebiotic utilization capacity can be expected in the members of the endogenous microbiome. In this context, not only the relative abundance of the endogenous microbiome members that can express such redundant functions and thereby compete for the same substrates, but also their substrate affinity and utilization rate relative to the administered *L. plantarum* strains will play a prominent role in the *in situ* selectivity of the *L. plantarum* fitness stimulation^[37-40]^. The *L. plantarum* intestinal fitness advantage induced by GOS or inulin supplementation was again assessed in rats that were fed a high-calcium control diet or the same diet supplemented with GOS or inulin. The mixture of the seven strains that was also used *in vitro* was gavaged (given once at the start of the experiment) in these rats and the population dynamics of these strains were followed up to 7 days post gavage in fecal samples. Strikingly, these experiments did not reveal any enrichment for any of the strains in the mixture, and the populations present in feces displayed a virtually identical strain composition compared to the gavaged inoculum. Nevertheless, the intestinal persistence of the *L. plantarum* community as a whole was significantly enhanced in rats that were fed a prebiotic-supplemented diet (GOS or inulin) compared to the un-supplemented control diet. Notably, consistent with earlier results, the prebiotic effects on persistence enhancement were more pronounced in high-calcium diets compared to low-calcium diets in these experiments^[41]^. These observations indicate that contrary to the strain-specific fitness benefit observed *in vitro*, the intestinal fitness of *L. plantarum* was enhanced by prebiotics in a strain-independent manner, irrespective of the prebiotic utilization phenotype of the individual strains.

Further investigation of the fecal samples obtained from these rats (prior- and post-gavage of the *L. plantarum* strain mixture) illustrated the prominent confounding effects of prebiotic supplementation, which elicited significant changes in the intestinal microbiome composition and increased the levels of fecal short chain fatty acids (SCFA) and other organic acids^[43]^. The effects of supplementation on the endogenous microbiome were highly similar between inulin and GOS, both leading to a marked increase in the relative abundance of *Bifidobacterium*. This increase was accompanied by a rise in the relative abundance of the *Faecalibaculum* genus, but only in diets high in calcium, not in those low in calcium^[41,43]^. The species- and strain-specific utilization of GOS and inulin has been well described in members of the *Bifidobacterium* genus^[44-47]^. Furthermore, the public genomes of *Faecalibaculum rodentium* encode secreted proteins with glycoside hydrolase (GH) family 32 domains that are typically involved in inulin utilization^[48]^. These genomes also encode lactose-PTS systems and genetically linked intracellular 6-P-β-galactosidases that resemble lactose-PTS systems found in *Lactobacillus gasseri* strains that were described to grow on GOS but lacked orthologues of the lactose/galactose-specific permease that is typically involved in lactose-import^[49,50]^. These highly abundant endogenous microbes probably outcompete the administered *L. plantarum* strains when it comes to prebiotic utilization as a substrate for growth. Nevertheless, the resulting intestinal milieu is apparently more favorable for the colonization of *L. plantarum*, which is probably related to the elevated levels of especially lactic and acetic acid in the high-calcium and prebiotic-supplemented diets compared to control diets, an environmental condition that agrees very well with the acidophilic character of *L. plantarum*^[41]^.

The experiments assessing the *L. plantarum* intestinal persistence as a function of prebiotic utilization, disrupts the synergistic synbiotic concept that was aimed for. The intestinal fitness advantage observed is independent of a direct interaction between the substrate and the microorganisms, but is achieved through the effect of the prebiotic substrate on the endogenous microbiome. Thereby, the synbiotic combinations of *L. plantarum* and prebiotics actually fall under the concept of complementary synbiotics, illustrating the complexity of accurately assessing the term “selectivity” in the synbiotic definition and discriminating between truly synergistic *vs*. complementary synbiotic delivery systems.

## PERSPECTIVE

The direct synergy between the probiotic and prebiotic components in a synergistic synbiotic represents a highly attractive concept. This is particularly based on the direct interaction of these two ingredients that enables their rational and experimentally verifiable design intended to adequately predict the *in situ* functionality when these products are delivered to the consuming host organism. However, the reviewed work above raises doubts about the reliability of such rational and experimentally driven design of synergistic synbiotics, which may eventually turn out to act as complementary synbiotics. Analogous to what is described for *L. plantarum*, competition experiments in GOS-fed gnotobiotic mice did not reveal a competitive advantage for *Limisilactobacillus reuteri* 6475 that is able to utilize this prebiotic substrate compared to its isogenic mutant-derivative that is unable to utilize GOS^[51]^. Nevertheless, irrespective of the precise nomenclature used for synbiotic concepts, the findings described above do support the usefulness of synbiotics in the intention to enhance the delivery of health-promoting microorganisms to their site of action (e.g., the human or animal intestinal tract). However, their selectivity in enhancing a specific microbial strain is unlikely to be achievable, especially when employing prebiotic substrates that are “commonly” degraded by endogenous microbiota members (e.g., GOS, FOS or inulin). Refinement of the substrate to chemically pure prebiotics rather than the current mixtures of prebiotic constituents such as IMOS and GOS (see above and ref^[29,32]^) may enhance the selectivity of such substrates in stimulating a specific strain or species. However, purification and/or chemical synthesis of these chemically pure prebiotics to generate more selective prebiotic substrates could be laborious and costly, which may compromise the economic feasibility of this scenario. Still, identifying more selective substrate compounds would provide more credible approaches to creating synergistic synbiotics compared to the canonically used prebiotics such as GOS, FOS, and inulin. In addition, selecting substrates that require an extensive enzymatic pathway for their degradation and utilization may contribute to the selectivity in stimulating only microorganisms that encode the entire enzyme repertoire required for substrate utilization. Besides the stimulation of the co-administered microorganism (i.e., the synergistic synbiotic concept), high specificity and selectivity substrates may also be very attractive compounds to stimulate an endogenous microbiota member in a precision-prebiotic approach. As an example, the endogenous *Bifidobacterium* population in the intestine was apparently selectively stimulated in healthy human volunteers by the consumption of synthetic human milk oligosaccharide (HMO) constituents 2’-fucosyllactose (2’FL) and/or Lacto-*neotetraose* (*LNnT*)^[52]^. However, and in view of the diversity of microbiome responses in individuals, one can question whether the *Bifidobacterium* stimulation is not simply the only common response in the participants rather than a truly selective response remains unclear, since additional microbiome responses may simply have remained undetected due to a lack of conservation among (i.e., distinct microbiome species reacting in individual participants prevents their detection in community-based microbiome response analyses) the participants. Extending this line of research toward chemically defined and pure prebiotics could determine to what extent certain single or mixed carbohydrate constituents of a prebiotic could selectively stimulate particular microbes *in situ*.

**Figure 2:**
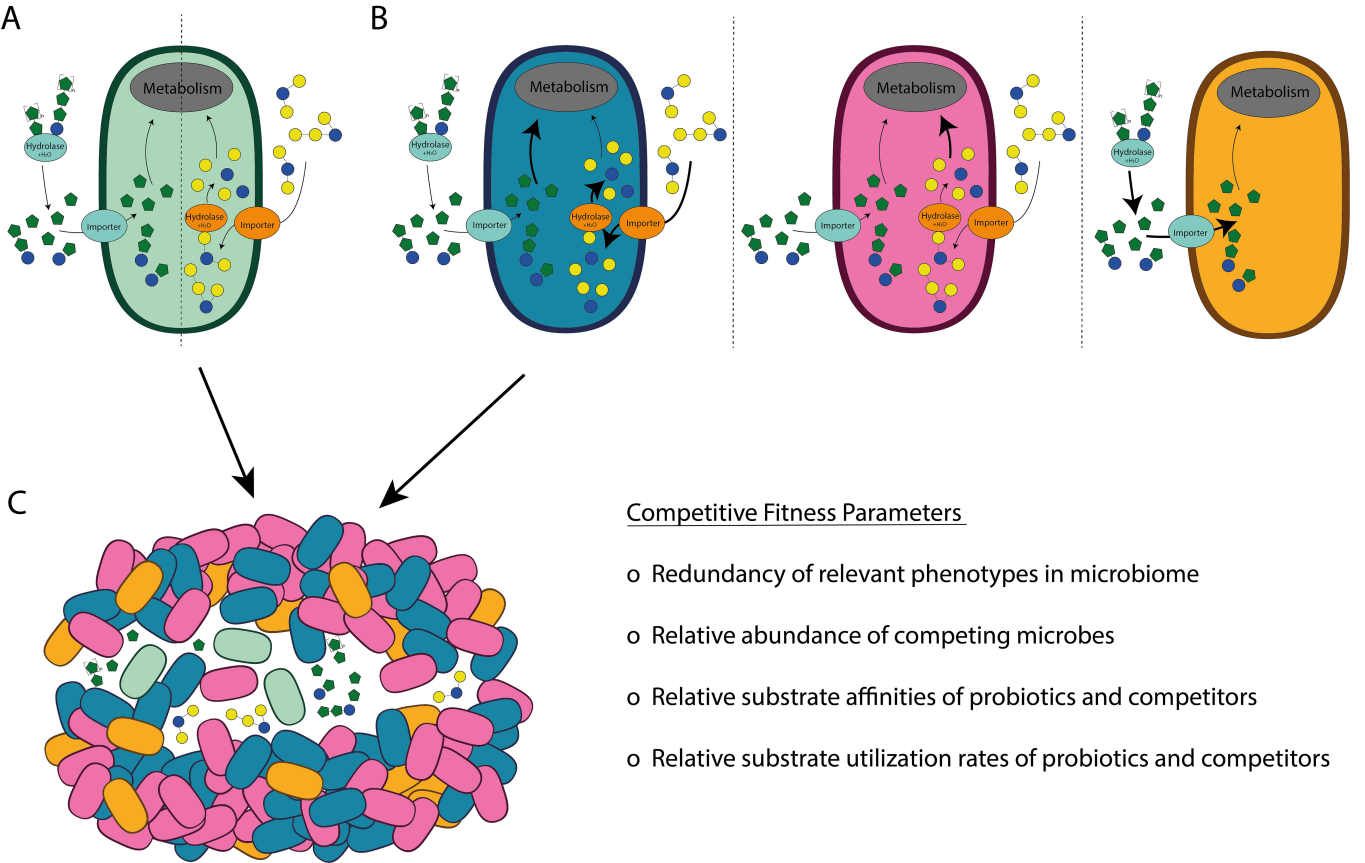
Competitive environmental fitness parameters that influence synbiotic mechanisms. (A) Exemplary prebiotic utilization strategies by a co-administered probiotic; left side of the cell: extracellular hydrolysis of the oligo- or polymeric substrate (e.g., FOS or inulin) with the subsequent import of released mono-saccharides or small oligosaccharides (i.e., sucrose and 1-kestose) that enter intracellular metabolism, with partial release of the substrate into the environment as “public goods”; right side of the cell: direct import of small oligosaccharides (e.g., tri- and tetra-saccharides in GOS) with subsequent intracellular hydrolysis and metabolization, i.e., “privatized goods”; (B) exemplary scenarios of endogenous microbiome members that can compete with the co-administered probiotic for the utilization of the prebiotic substrates. These competing capacities may be redundantly present in multiple members of the microbiome. Moreover, these redundant utilization pathways may have varying affinities and utilization rates for the prebiotic substrates, indicated by variations in arrow thickness; (C) In addition to the microbiome’s redundancy in prebiotic utilization capacities, their variable substrate affinities and utilization rates, the high relative abundance of competing microbes in comparison to the introduced probiotic favors complementary synbiotic effects rather than synergistic synbiotic effects, especially when the prebiotic substrate can readily be utilized by multiple members in the ecosystem and thus has poor species- or strain-specific selectivity. Consequently, the competing capacity of the dominating members of the microbiome (blue, pink, and orange) prevents the utilization of the prebiotic by, and the resulting growth stimulation of, the co-administered probiotic (green). FOS: Fructo-oligosaccharides; GOS: galacto-oligosaccharides.

Irrespective of their use as part of a synergistic synbiotic mixture or as precision prebiotic, the proposed substrate compounds and their claims toward selectivity should be rigorously evaluated using robust *in vivo* nutrient-competition models to ensure that observed fitness enhancement effects are driven by direct interaction between substrate and microorganism. Experimental verification of the selectivity of synergistic synbiotics or the precision prebiotic requires that ecosystem interaction and nutrient competition concepts are taken into account and controlled for, e.g., “cooperative traits” and their role in generating “public goods”, the substrate monopolization as “personalized goods”, and the nutrient competition capacity redundancy and abundance, as well as the corresponding substrate affinity and utilization rates [Figure 2].

How do the arguments raised above influence our perspective on the spectacular health effects of the administration in newborn infants of *L. plantarum* combined with FOS synbiotic, reported by Panigrahi *et al.*^[8]^? The administration regiment employed in that study provides this synbiotic to infants during their first weeks of life, which was demonstrated to achieve the engraftment of the *L. plantarum* strain in the intestine of these infants for a period of at least several months^[8,9]^. With such an administration regimen, the FOS prebiotics are highly unlikely to modify the intestinal milieu for such an extended period, implying that the engraftment observed is probably strongly related to the relatively empty niche of the GI tract of newborns that the *L. plantarum* strain is introduced into, which may explain the strain’s capacity to colonize and occupy this niche for an extended period. These arguments support the hypothesis that administration of the *L. plantarum* strain alone (without the prebiotic FOS) would likely achieve similar results. However, these studies did not include such a control group and, therefore, are unable to establish the importance of FOS in this approach. Notably, experiments that employed the simultaneous administration of *L. plantarum* Lp900 and inulin could enhance gastric survival (i.e., by 1-2 logs) but failed to affect intestinal persistence over an extended period^[12]^. Analogously, *in vitro* studies that stimulated conditions of the gastrointestinal tract demonstrated that *L. plantarum* survival during gastric passage (i.e., the acidity barrier of the stomach) could be significantly enhanced by the presence of glucose during the gastric transit (in concentrations as low as 0.05% w/w), but no such effect was achieved by the presence of the unfermentable enantiomer of glucose (i.e., L-glucose), indicating that the cellular energy state is crucial for this enhanced gastric stress tolerance^[53]^. These findings may imply that the co-administration of FOS with *L. plantarum* ATCC202195 employed by Panigrahi *et al*.^[8]^ may have enhanced the survival of the probiotic strain during gastric passage by energizing the probiotic strain based on the use of the low-DP constituents of this prebiotic (i.e., fructose, sucrose, and 1-kestose, see above^[12,29,41]^), supporting more effective *in situ* delivery in the infant gut. Therefore, the administration of FOS could have influenced the outcome of the trial by improving the gastric survival and intestinal delivery of the probiotic, but this effect is probably not specific for FOS and is likely to be also achieved with a simple sugar such as glucose. In conclusion, it remains unclear to what extent this landmark study should be regarded as a true synbiotic study or could actually better be seen as a probiotic study, because it seems likely that the effect of the co-administered FOS simply depends on the energy state of the probiotic rather than any form of selective fitness advantage.

The above-mentioned concept of precision prebiotics is particularly relevant in view of the advances in our knowledge of the human intestinal microbiota. Traditional prebiotic applications have commonly targeted the stimulation of the endogenous populations of members of the *Bifidobacterium* genus or the *Lactobacillaceae* family based on their associations with health benefits. Precision prebiotics may offer opportunities to selectively stimulate the growth of other health-promoting members of the endogenous microbiome^[54]^. As an example, high abundance of *Faecalibacterium prausnitzii* (*F. prausnitzii*) has been inversely associated with flare incidence in inflammatory bowel disease (IBD) patients, and specific strains of the species (A2-165 and HTRF-F) were shown to alleviate inflammation in murine colitis models^[55,56]^. Moreover, *F. prausnitzii* is one of the main butyrate producers in the intestine^[57]^, an essential short chain fatty acid involved in the regulation of a wide spectrum of health-promoting effects (e.g., trans-epithelial fluid transport, amelioration of mucosal inflammation, reinforcement of epithelial defense barriers, and intestinal motility)^[58]^. Strains of *F. prausnitzii* display a high degree of genetic diversity, particularly reflected in diverse carbohydrate utilization and immunomodulatory phenotypes^[59]^, suggesting their strain-specific response to prebiotic supplementation. Extending the characterization of the genomic and phenotypic diversity of strains belonging to this species could offer new avenues toward the application of the species as candidates for next-generation probiotics^[60]^. Intriguingly, both FOS and inulin were shown to have stimulatory effects on *F. prausnitzii* in humans^[61,62]^, and the capacity to utilize the substrate was confirmed for *F. prausnitzii* strain A2-165 *in vitro*^[63]^. Similarly, there is also evidence that GOS consumption can stimulate endogenous *F. prausnitzii* populations in humans^[64,65]^, although GOS could not effectively support the growth of *F. prausnitziii* A2-165 *in vitro,* which led to the suggestion that GOS-mediated stimulation of *F. prausnitzii* would depend on bifidobacterial degradation of GOS and cross feeding^[63,66]^. However, the lack of growth of *F. prausnitzii* A2-165 is potentially meaningless in explaining the stimulatory effect of GOS supplementation on the relative abundance values of the endogenous populations of *F. prausnitzii* in humans, which may simply reflect the high diversity in carbohydrate utilization capacities in the strains of this species^[60]^. The genomic information available for this species is rapidly expanding, and at present, more than 700 *F. prausnitzii* genomes are available at NCBI [747 *F. prausnitzii* genome assemblies, including a large amount of metagenome-assembled genomes (MAGs), in July 2024]. Analogous to what is described above for *L. plantarum*, *F. prausnitzii* prebiotic matchmaking screening combined with detailed substrate utilization analysis by HPAEC-PAD and UPLC-MS can generate data that enable gene-trait matching to identify the genetic loci in *F. prausnitzii* that are required for the utilization of specific prebiotic constituents. Knowledge about the *F. prausnitzii* genes required for utilization of specific prebiotic constituents, offers the possibility of predicting which prebiotic substrates could selectively stimulate an individual’s endogenous *F. prausnitzii* populations by metagenome mining for its carbohydrate utilization genes. In addition, following the further development of *F. prausnitzii* strains as next-generation probiotics, the acquired knowledge could facilitate the design of synergistic synbiotics to enhance the delivery of these bacteria to the intestine in a viable and metabolically active form, which would stimulate their engraftment and persistence. Similar to what is described for *L. plantarum*, evidencing the selectivity of *F. prausnitzii* stimulation is crucial; this can be facilitated by the expansion of our knowledge about the specific genes involved in the utilization of specific substrates. Such efforts would build toward the concept of precision prebiotics, as well as next-generation probiotic-containing synergistic synbiotics.

## CONCLUSION

Although the research track using *L. plantarum* started with the ambition to design synergistic synbiotics for specific strains of this species, it ended up with complementary synbiotics that could enhance the intestinal delivery and persistence of *L. plantarum* in a general rather than a strain-specific manner. The generation of convincing evidence for the selectivity of the direct interaction between the substrate and the microorganism that are combined in synergistic synbiotics requires dedicated *in vivo* competition experiments. Such experiments should include the analysis of potential confounding factors that may disrupt the “synergistic” characteristic of the synbiotic. Part of these confounding effects can be anticipated by taking ecosystem interaction and nutrient competition mechanisms [Figure 2] into account and specifically addressing them in the measurements included in the experimental design.

The concept of precision prebiotics using highly defined and pure prebiotic compounds (rather than the current substrate mixtures) combined with identification of the genes required for their utilization, offers a promising approach toward metagenomics-based, and thereby individualized selective stimulation of endogenous members of the microbiome that are associated with a healthy host. Moreover, a similar genome-based precision-substrate matching approach can support the design of truly selective synergistic synbiotics, containing either the classical *Bifidobacterium* and *Lactobacillaceae* probiotics or the emerging next-generation probiotics. A substantial amount of research will be required to progress our knowledge to a level that would enable these strategies. Additionally, it should be realized that profitable bulk production of the eventually identified precision prebiotic substrates in a chemically pure form may be challenging.

## DECLARATIONS

### Copyright

© The Author(s) 2024.
